# Molecular Crosstalk Between RUNX2 and HIF-1α in Osteosarcoma: Implications for Angiogenesis, Metastasis, and Therapy Resistance

**DOI:** 10.3390/ijms26157642

**Published:** 2025-08-07

**Authors:** Anuja Gajanan Magar, Vivek Kumar Morya, Kyu-Cheol Noh

**Affiliations:** 1 School of Medicine, Hallym University, Chuncheon-si 24252, Republic of Korea; 2 Hallym University Sacred Heart Hospital, Anyang-si 14068, Republic of Korea

**Keywords:** osteosarcoma, RUNX2, HIF-1α, angiogenesis, metastasis, therapy resistance

## Abstract

Runt-related transcription factor-2 (RUNX2) is an integral player in osteogenesis and is highly expressed in osteosarcoma. Emerging evidence suggests that aberrant RUNX2 expression is a key factor in osteosarcoma oncogenesis. Patients with advanced stages of osteosarcoma overexpressing RUNX2 are more likely to have high tumour grades, metastasis, and lower overall or progression-free survival rates. Thus, RUNX2 is considered a potential candidate for targeted therapy of osteosarcoma. Hypoxia-inducible factor-1α (HIF-1α) is a key transcription factor involved in the regulation of cellular reprogramming in response to hypoxia. Overexpression of HIF-1α decreases overall survival, disease-free survival, and chemotherapy response and promotes tumour stage and metastasis. Hence, our review focused on highlighting the intricate network between RUNX2 and HIF-1α, which support each other or may work synergistically to develop resistance to therapy and osteosarcoma progression. An in-depth understanding of these two important tumour progression markers is required. Therefore, this review focuses on the role of RUNX2 and HIF-1α in the alteration of the tumour microenvironment, which further promotes angiogenesis, metastasis, and resistance to therapy in osteosarcoma.

## 1. Introduction

**Figure 1:**
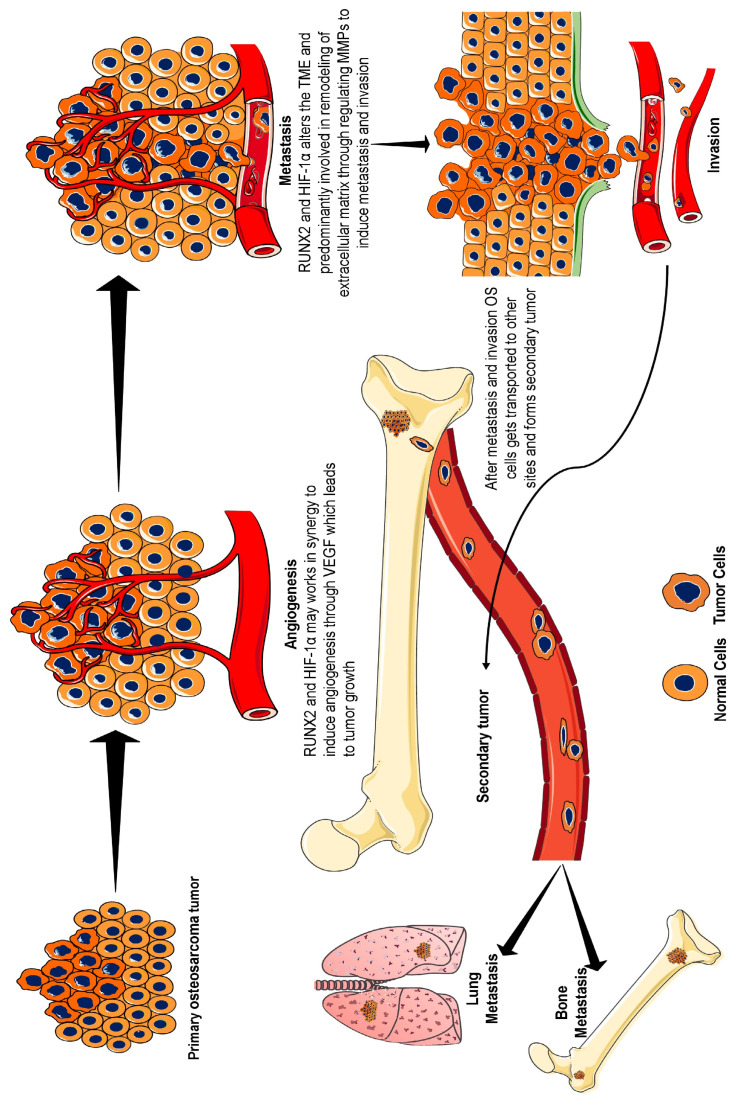
Development and progression of osteosarcoma. Under hypoxic conditions, HIF-1α is induced and creates a tumour-friendly environment through aberrant angiogenesis via VEGF signalling. RUNX2 also plays an active role, or it may be that both of them work in synergy to stabilize VEGF, an angiogenesis precursor. Furthermore, HIF-1α and RUNX2 alter the tumour microenvironment through enhanced glycolysis and induce MMP expression, leading to extracellular matrix remodelling. MMP causes metastasis and invasion, which helps cancer cells to transport to other bone and lung sites. In osteosarcoma, the primary site of metastasis is the lung, followed by the other bones. Therefore, the overexpression of RUNX2 and HIF-1α can be considered a biomarker for osteosarcoma because of their roles in the stimulation of angiogenesis, metastasis, and invasion. Abbreviations: HIF-1α, hypoxia-inducible factor-1α; RUNX2, runt-related transcription factor-2; VEGF, vascular endothelial growth factor; MMP, matrix metalloproteinase.

Osteosarcoma is a bone tumour that arises in the metaphysis of the long bones adjacent to the growth plate [1]. Osteosarcoma (OS) is one of the most aggressive bone tumours and accounts for 56% of all bone sarcomas. The distal femur around the knee joint produces two-thirds of these, whereas the proximal tibia and proximal humerus follow suit. It primarily affects children, teenagers, and young adults, with a median age of 16 years [2,3]. Every year, approximately 4.4 cases of children are diagnosed with osteosarcoma [4]. However, a second peak of incidence (10% of cases) occurs in adults over 60 years of age and is frequently linked to Paget’s disease of the bone [5,6,7]. Approximately 25% of osteosarcoma patients have already metastasized during diagnosis. Most cases (85%) had lung metastases, 8–10% of cases had bone metastases, and 8% had both lung and bone metastases [6,8]. This suggests that the lungs are the most common site of metastatic spread in individuals with osteosarcoma (Figure 1) [6]. This evidence suggests that metastasis is a primary concern in osteosarcoma [9]. For patients with localized tumours, the five-year survival rate exceeds 70%; however, for those with distant metastases or recurrent ailments, the survival rate drops substantially to <30% [10]. These results show that the survival rates have not increased over the past three decades. However, owing to advancements in therapies, the incidence rate has remained mostly steady, but the mortality rate has declined somewhat [4].

Runt-related transcription factor-2 (RUNX2) is a crucial regulator of the transcriptional activation of signalling pathways involved in chondrogenic and osteogenic processes [11,12]. However, in osteosarcoma, RUNX2 has been found to be dysregulated and abruptly expressed [13]. These factors have a significant impact on the development and evolution of osteosarcoma, as they are predominantly involved in the regulation and development of bones. RUNX2 plays a crucial role in osteosarcoma by stimulating angiogenesis, promoting metastasis and proliferation, enhancing cancer stemness, and contributing to drug resistance [14,15,16]. Moreover, tumour progression necessitates cellular adaptation to low-oxygen conditions, a process mainly driven by hypoxia-inducible factor-1α (HIF-1α), which orchestrates the expression of hypoxia-responsive genes [17]. In solid tumours, metabolic alterations and stimulation of glycolytic factors are observed in the presence of HIF-1α, which helps to create a tumour microenvironment. In such a tumour microenvironment, tumour-suppressive genes are downregulated, which helps activate oncogenes to support tumour growth [18,19]. Similarly, HIF-1α promotes metabolic alterations that aid in the progression of osteosarcoma, and its elevated expression is more likely to result in poor clinicopathological traits, advanced cancer stage, metastasis, low survival, and resistance to chemotherapy [19,20,21].

Thus, there may be an intricate relationship between RUNX2 and HIF-1α that allows them to complete each other or may work synergistically to develop resistance to therapy and promote osteosarcoma progression (Figure 1). Therefore, this study emphasizes the significance of HIF-1α and RUNX2 in osteosarcoma and reinforces the need to regulate these two vital factors to establish an effective osteosarcoma treatment. This new approach of considering RUNX2/HIF-1α as an integral transcription factor in osteosarcoma may aid in mitigating tumour development, improving treatment resistance, and accelerating the overall survival rate of patients with osteosarcoma.

## 2. Molecular Cross-Talk Between RUNX2 and HIF-1α

As discussed previously, HIF-1α alters the tumour microenvironment and promotes angiogenesis and glycolysis, whereas RUNX2 is an important bone transcription factor. Both are highly expressed in osteosarcoma and their presence results in osteosarcoma progression. Thus, it can be postulated that there may be an intricate relationship between these two factors that complement each other or may work synergistically in osteosarcoma.

### Role of RUNX2 and HIF-1α in Osteogenic and Angiogenic Signalling Pathways

HIF-1α protein levels are regulated by oxygen-dependent degradation mechanisms. Under normoxic conditions, specific proline residues near the C-terminal region are hydroxylated by prolyl hydroxylase enzymes, which function as oxygen sensors. This hydroxylation enables HIF-1α to interact with von Hippel-Lindau (VHL) protein, a component of the E3 ubiquitin ligase complex, marking it for ubiquitination and subsequent degradation via the proteasome. In contrast, during hypoxia, this hydroxylation process is impaired, leading to the stabilization and accumulation of HIF-1α. The stabilized protein translocates to the nucleus, where it dimerises with the constantly expressed HIF-β subunit, also referred to as (aryl hydrocarbon receptor nuclear translocator) [22]. This α/β heterodimer then binds to hypoxia-response elements (HREs) in the promoter regions of target genes involved in angiogenesis such as VEGF, erythropoietin, and TGF-β3. The activation of these genes facilitates neovascularization, osteogenesis, and tumour development (Table S1) [22,23,24].

RUNX2 is initially active in mesenchymal condensation during limb development and is expressed throughout the skeletal formation. In addition to its role in bone development, RUNX2 is transiently present in vascular endothelial and smooth muscle cells during blood vessel formation, indicating its possible function in angiogenesis [23]. Studies have shown that RUNX2 physically and functionally interacts with HIF-1α to modulate VEGF production. Specifically, RUNX2 and HIF-1α co-localize within distinct nuclear compartments and interact via the runt domain of RUNX2. Chromatin immunoprecipitation (ChIP) analyses confirmed their binding to the chromatin of the VEGF-A promoter region (Table S1) [23,24,25]. This evidence suggests that their cooperative action enhances VEGF expression, and that both proteins are simultaneously present in hypertrophic chondrocytes and osteoblasts.

In hypertrophic wild-type chondrocytes, simultaneous expression of RUNX2 and HIF-1α was correlated with elevated vascular density. However, in RUNX2-deficient mice, both HIF-1α levels and vascularisation within the tibial growth plate are markedly diminished, underscoring the essential role of RUNX2 in regulating angiogenesis during endochondral bone development. Mechanistically, RUNX2 binds to the oxygen-dependent degradation domain (ODDD) of HIF-1α, thereby competing with the von Hippel–Lindau (VHL) protein and promoting HIF-1α stabilization. RUNX2-mediated stabilization of HIF-1α enhances its expression and angiogenic potential in vitro, as evidenced by increased VEGF promoter activity, elevated VEGF secretion, and enhanced tube formation in human umbilical vein endothelial cells (HUVECs) [25,26].

As mentioned earlier, RUNX2 and HIF-1α co-localize in the nuclei of preosteoblasts and co-occupy the VEGF promoter region, synergistically enhancing VEGF transcription. However, RUNX2 knockdown impaired HIF-1α induction and worsened glycolytic metabolism, whereas HIF-1α knockdown suppressed glycolysis in chondrocytes. Even under normoxic conditions, RUNX2 transcriptionally regulates HIF-1α by binding to its promoter and promoting its nuclear translocation. RUNX2 prevents HIF-1α degradation by blocking ubiquitination. Notably, this interaction does not rely on canonical RUNX2 binding motifs; instead, RUNX2 amplifies HIF-1α-driven transcription of VEGF and other HRE-responsive genes under both normoxic and hypoxic conditions [24,25,26].

RUNX2 overexpression in malignancies such as osteosarcoma contributes to increased VEGF production and enhances anti-apoptotic signalling within hypoxic microenvironments. RUNX2 is often upregulated in cancers, such as osteosarcoma, colon cancer, prostate cancer, thyroid cancer, and melanoma, underscoring its oncogenic role. It also transactivates several tumour-promoting genes, including survivin, MMP-2, MMP-9, and VEGF, thereby linking it to cancer progression, metastasis, and angiogenesis [24]. Under hypoxic conditions, HIF-1α accumulates owing to its oxygen-sensing capability, promoting tumour development through VEGF-dependent angiogenesis. Consequently, the hypoxic HIF-1α/VEGF regulatory axis is crucial for tumour progression. Notably, RUNX2 enhances HIF-1α-mediated VEGF induction, and recent studies suggest that RUNX2 may also contribute to drug resistance in malignant tumours [27,28].

## 3. RUNX2–HIF-1α Role in Osteosarcoma Progression

RUNX2 can directly upregulate HIF-1α transcription and stabilize it by protecting it from VHL-mediated degradation [24]. This positive feedback loop is particularly pronounced under PI3K/AKT-driven angiogenic signalling, which is often hyperactive in osteosarcoma. Therefore, it represents a promising therapeutic target. Dual HIF-1α/RUNX2 inhibitors have been proposed to disrupt the VEGF pathway, whereas isoform-specific HIF blockade offers the potential to enhance specificity and bypass adaptive resistance mechanisms [29].

### 3.1. Tumour Microenvironment

**Figure 2:**
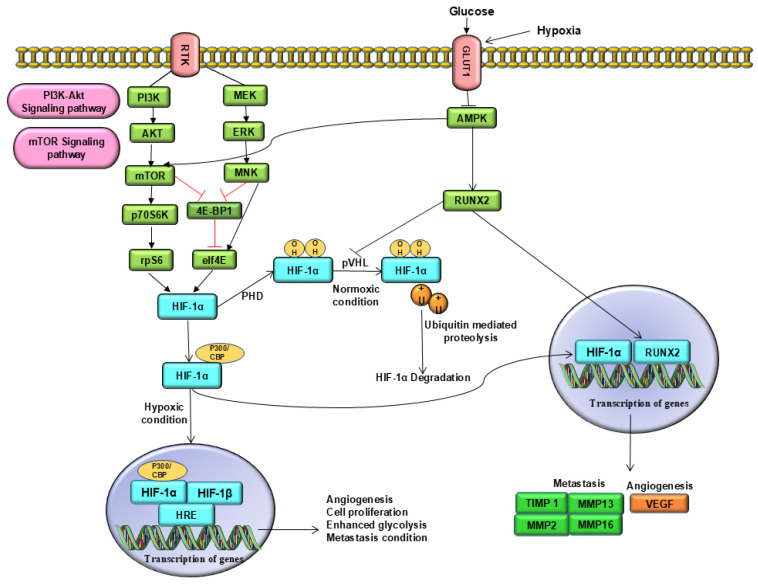
Molecular crosstalk between RUNX2 and HIF-1α in osteosarcoma. Under normoxic conditions, HIF-1α is hydroxylated by PHDs. PHDs hydroxylate proline residues and provide a binding site for pVHL, a tumour suppressor protein. This tumour suppressor gene leads to polyubiquitination and proteasomal degradation of HIF-1α. This hydroxylation impedes the binding of HIF-1α to the transcriptional coactivators p300 and CBP. In contrast, under hypoxic conditions, PHDs are unable to hydroxylate HIF-1α. Therefore, HIF-1α translocates to the nucleus and dimerises with HIF-β subunit. This complex activates the HIF pathway and promotes the transcription of genes that enhance glucose metabolism, angiogenesis, metastasis, and invasiveness. In addition, RUNX2 competes with the E3 ubiquitin ligase pVHL to prevent the degradation of HIF-1α. This stabilized expression of HIF-1α further enhances glycolysis via overexpression of GLUT1 receptors and enhances glucose transport. This will aid in the metabolic shift towards aerobic glycolysis and support rapid tumour growth. Additionally, the activity of AMPK, a negative regulator of mTOR, and RUNX2 is suppressed under hypoxic conditions, leading to the stabilization of the mTOR pathway responsible for cell proliferation. Downregulation of AMPK prevents degradation and helps in RUNX2 stabilization. This synergistic interaction between RUNX2 and HIF-1α induces VEGF expression and promotes angiogenesis, metastasis, and invasion. Abbreviations: AMPK, adenosine monophosphate-activated protein kinase; CBP: Core binding protein; GLUT1, glucose transporter 1; HIF-1α, hypoxia inducible factor-1α; mTOR, mammalian targets of rapamycin; PHDs, prolyl hydroxylase domain; PI3K/AKT: Phosphatidylinositol 3-kinase/Protein kinase B; RUNX2, runt-related transcription factor 2; pVHL, von Hippel-Lindau protein; VEGF, vascular endothelial growth factor.

Glucose uptake promotes osteoblast differentiation by preventing AMPK-mediated proteasomal degradation of RUNX2, and supports bone formation by modulating other AMPK-related pathways [30]. Activation of the AMPK pathway is responsible for proteasomal degradation and RUNX2 [31]. However, it is well known that hypoxic conditions or activation of HIF-1α increase glucose concentration, aiding cancer cell proliferation [22]. RUNX2 overexpression and AMPK pathway downregulation have been observed [32]. Similar findings have been reported in breast cancer models, in which RUNX2 knockdown activated AMPK and suppressed tumour progression [33]. Nevertheless, higher intracellular glucose levels suppressed AMPK activity. For instance, GLUT1-mediated glucose transport suppresses AMPK activity and activates mammalian target of rapamycin (mTOR), another signalling pathway regulated by AMPK. mTOR activation enhances cellular proliferation and promotes tumour progression [34]. mTOR is a positive regulator of HIF-1α and drives its synthesis in a multifaceted manner via 4E-BP1/eIF4E (Figure 2) [35]. mTOR activation leads to more vascularised tumours by activating HIF-1α-induced VEGF signalling. For example, canagliflozin treatment decreased metastasis, angiogenesis, and metabolic reprogramming in hepatocellular carcinoma by inhibiting HIF-1α protein accumulation, likely by targeting the AKT/mTOR pathway [36]. Therefore, there may be a feed-forward mechanism between RUNX2 and GLUT-1, where RUNX2 binds to the GLUT1 promoter region, thereby increasing GLUT1 transcription. This high intracellular glucose level prevents proteasomal degradation of RUNX2 by suppressing AMPK activity and promoting HIF-1α activation (Figure 2) [37]. As observed in osteoarthritis studies, RUNX2 activation governs HIF-1α expression by linking it to its promoter region, significantly improving glucose absorption and ATP production [26]. Conversely, RUNX2 deficiency inhibits HIF-1α activation and exacerbates chondrocyte degeneration due to a lack of glucose [25]. Thus, it can be concluded that RUNX2 and HIF-1α enhance glucose uptake to prevent their degradation. Elevated glucose levels also create a tumour microenvironment for osteosarcoma cells to survive, promote metastasis, and develop resistance to chemotherapy, resulting in a low survival rate [33,38].

### 3.2. Angiogenesis

HIF-1α is a well-known factor that promotes angiogenesis by regulating angiogenic stimulators such as VEGF, platelet-derived growth factor, and transforming growth factor beta [24,25,26,39,40]. In hypoxic environments, osteosarcoma cells exhibit high levels of HIF-1α and VEGF, which promote angiogenesis. According to a meta-analysis, HIF-1α overexpression correlates with poor patient outcomes, making it a prognostic biomarker for predicting patient outcomes [39,40]. Notably, RUNX2 and HIF-1α enhance the expression of angiogenic genes, thereby supporting angiogenesis (Table S2) [23]. As previously mentioned, under tumour conditions, RUNX2 overexpression stabilizes HIF-1α and induces the expression of VEGF, an inflammatory mediator of angiogenesis [24,25,26,41]. Similar findings indicate that RUNX2 overexpression in mesenchymal cells upregulates HIF-1α and VEGF expression [42]. Conversely, silencing HIF-1α reduces the expression of the target protein VEGF and decreases RUNX2 expression (Table S1) [43]. Thus, under hypoxic tumour conditions, RUNX2 overexpression enhanced VEGF expression, promoted anti-apoptotic activity, and accelerated tumour progression (Table S1). Moreover, RUNX2 stabilizes HIF-1α by inhibiting its degradation via the ubiquitin–proteasome system (UPS) pathway, as previously noted [26]. It also promotes angiogenesis in endothelial cells through glucose-induced phosphorylation and plays a key role in the progression and malignant transformation of chondrosarcoma by elevating VEGF expression. In melanoma, the Runt domain of RUNX2 serves as a critical regulator of neoangiogenesis, driving the formation of new blood vessels [42]. Consequently, RUNX2 and HIF-1α synergistically promote angiogenesis, a vital process in the tumour microenvironment that provides nutrients and oxygen to the growing tumour. HIF-1α induces VEGF and other proangiogenic factors, whereas RUNX2 amplifies this effect, ensuring robust blood vessel formation. This evidence underscores the molecular crosstalk between RUNX2 and HIF-1α in tumour conditions, leading to disease progression through aberrant angiogenesis, resulting in tumour vascularization and poor prognosis. For instance, in bone metastasis, a hypervascularised tumour microenvironment supports tumour expansion, nutrient delivery, and metastatic dissemination. Importantly, RUNX2′s ability to stabilize HIF-1α and amplify angiogenesis in normoxic regions highlights its role in reshaping the bone niche to favour tumour colonization. Targeting the RUNX2–HIF-1α interaction or its downstream angiogenic mediators holds promise for curbing tumour-induced neovascularization and impeding metastatic progression within bone tissue (Table S1).

### 3.3. Metastasis

The extracellular matrix remodelling is a crucial process for metastasis through the regulation of matrix metalloproteinases (MMPs), such as MMP-9, MMP-13, and MMP-16. These are major factors associated with metastasis. MMP causes the degradation of the extracellular matrix, making osteosarcoma cells more prone to metastasis [44,45]. In osteosarcoma, upregulation of MMP-2, MMP-9, and MMP-13 has been observed in the presence of RUNX2, which promotes metastasis and invasion (Table S2) [46,47]. Similarly, Rici et al. reported that RUNX2 controls the transcription of MMP13, which contributes to oncogenesis and facilitates metastasis through transcription factor activator protein-1 [48]. Moreover, the pathways involved in cell adhesion and motility are regulated by RUNX2, which leads to a more aggressive osteosarcoma phenotype [16]. Won et al. reported that 23% (11 out of 48) of osteosarcoma tissue cores exhibited high RUNX2 expression, which was significantly associated with metastasis and showed a tendency toward poorer survival compared to the 60% (29 out of 48) with low expression (Table S2) [49]. Furthermore, positive RUNX2 staining was found in 60% (12/20) of biopsy samples and 73% (8/11) of metastatic tumour samples from osteosarcoma patients [50]. However, the silencing or downregulation of RUNX2 prevents metastasis. For example, in an osteosarcoma cell line study, RUNX2 silencing inhibited invasion capacity by suppressing VEGF, MMP-2, and MMP-9 expression (Table S2) [44]. Furthermore, HIF-1α supports this metastatic process by remodelling the vasculature and promoting epithelial–mesenchymal transition (EMT), a key step in metastasis [51]. causes the loss of epithelial cell polarity, weakening of cell-to-cell junctions, and reorganization of cytoskeletal proteins, which collectively promote tumour invasion and metastasis. HIF-1α suppresses E-cadherin expression while increasing the levels of Twist-related protein 1 [52]. Additionally, notable changes in the expression of VEGF, E-cadherin, and N-cadherin in osteosarcoma cells have been observed to be correlated with HIF-1α levels. These changes significantly enhance the migratory and invasive abilities of the cells [53]. Therefore, HIF-1α plays a key role in promoting or maintaining the metastatic and invasive behaviours of osteosarcoma cells [51,53]. Together, RUNX2 and HIF-1α collaboratively enhance the metastatic and invasive potential of osteosarcoma, where RUNX2 upregulates MMPs and other enzymes to degrade the extracellular matrix and HIF-1α supports this process through EMT (Table S3).

### 3.4. Chemotherapy Resistance

Osteosarcoma often exhibits elevated levels of RUNX2 protein and mRNA16. Consequently, RUNX2 overexpression is regarded as a hallmark of osteosarcoma, with chemoresistant tumours displaying more aberrant RUNX2 expression than non-resistant tumours. Although the underlying mechanism remains unclear, it has been found to RUNX2 contributes to p53 suppression and reduced chemotherapy responsiveness. A recent study suggested that elevated RUNX2 expression, along with the suppression of RUNX2/pAKT signalling, may reactivate p53 function and increase the sensitivity of osteosarcoma cells to apoptosis [54,55]. For example, in a quantitative expression analysis, Sadikovic et al. assessed the relative expression levels of 16 genes as potential biomarkers of osteosarcoma oncogenesis and chemotherapy response in a panel of 22 osteosarcoma tumours. RUNX2 is the only gene significantly overexpressed in osteosarcoma with a poor chemotherapy response (Table S2) [56].

**Figure 3:**
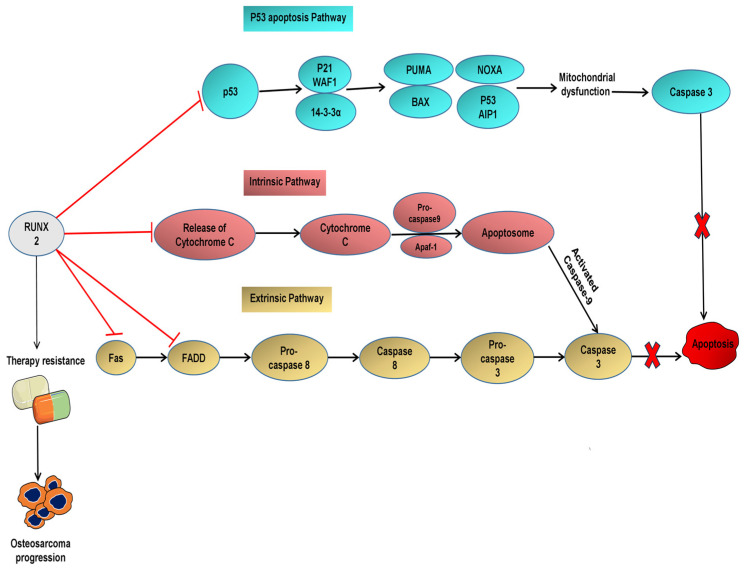
The role of RUNX2 in the development of therapy resistance RUNX2 prevents osteosarcoma cells from apoptosis by suppressing the activity of caspases and p-53 targeted genes. For example, RUNX2 downregulates the activation of p53-targeted genes including p21, WAF1, PUMA, and NOXA, which are responsible for apoptosis. In addition, overexpression of RUNX2 prevents the release of the extrinsic ligand Fas, adaptor protein FADD, and intrinsic apoptotic marker cytochrome C. It prevents the activation of caspase-3, caspase-8 and caspase-9. Thus, RUNX2 inhibits the intrinsic and extrinsic apoptotic pathways and protects osteosarcoma cells from apoptosis after chemotherapy. This implies that the regulation of RUNX2 in osteosarcoma will help enhance chemosensitivity and reactivate the apoptosis pathway. Abbreviations: Apaf-1: Apoptotic Protease Activating Factor 1; Bax, BCL-2-associated X protein; Fas, FS-7-associated surface antigen; FADD, Fas-associated death domain protein; NOXA, Phorbol-12-myristate-13-acetate-induced protein 1; PUMA: p53 upregulated modulator of apoptosis; RUNX2: runt-related transcription factor 2; WAF1: Wild-type Allele Frequency 1.

This suggests that RUNX2 is a crucial factor in protecting osteosarcoma cells from apoptosis [16]. Members of the p53 family of well-known tumour suppressor genes are pivotal for inducing apoptosis [57,58]. However, p53 mutations are prevalent in most osteosarcoma patients, possibly because of the inverse relationship between p53 and RUNX2 (Table S2) [13]. RUNX2 has also been identified as a negative regulator of p53 in response to DNA damage, as its forced expression inhibits apoptosis by downregulating p53 activity [59,60]. Similarly, stabilizing p53 through the MDM2 inhibitor nutlin-3 downregulates RUNX2 expression [13]. Conversely, RUNX2 knockdown enhances apoptosis mediated by adriamycin and the expression of p53-targeted genes (Table S2) [59]. Additionally, RUNX2 collaborates with HDAC6 to inhibit DNA damage-induced transcriptional and pro-apoptotic activities of p5359. Beyond p53, RUNX2 also impairs the transcriptional activity of other pro-apoptotic proteins such as TAp73 and TAp63 (Table S2) [61]. However, RUNX2 suppression reverses TAp63 activity and facilitates the induction of p53/TAp63-target genes responsible for cell death, such as p21WAF1, PUMA, and NOXA (Table S2) [62]. Numerous studies have demonstrated that miRNAs are aberrantly expressed in osteosarcoma and play direct or indirect roles in controlling the expression of RUNX2. Such as miR-150, enhanced anti-tumour properties and increases the sensitivity to chemotherapy by suppressing RUNX2 expression [55]. Moreover, the tumour suppressor MiR-203 reduced osteosarcoma cell growth and invasion and promoted apoptosis by enhancing chemosensitivity to cisplatin therapy by targeting RUNX2 [63]. Similarly, Lin et al. (2017) discovered that by suppressing RUNX2 expression, miR-203 reduced osteosarcoma cell growth and invasion and enhanced apoptosis (Table S2) [64]. These studies highlight that RUNX2 prevents apoptosis by suppressing the apoptotic pathway. For example, in mouse and human osteosarcoma cell lines, RUNX2 knockdown increased the release of extrinsic ligand FAs, adaptor protein FADD, and intrinsic apoptotic marker cytochrome C (Figure 3). Activation of these markers increases the susceptibility of cells to doxorubicin-induced apoptosis and improves tumour necrosis rate [16]. This implies that RUNX2 inhibits the intrinsic and extrinsic apoptosis pathways and reduced RUNX2 levels enhance chemosensitivity (Table S2). Therefore, RUNX2 is also a known marker of chemoresistance in osteosarcoma [16].

Along with RUNX2, HIF-1α is also an important and highly specific transcriptional regulator that is correlated with poor prognosis and the development of chemotherapy resistance [65]. For example, Xu et al. showed an increased antitumour effect of methotrexate and cisplatin in an in vivo model along with an HIF-1α inhibitor. Similar effects have been observed in cells isolated from chemoresistant patients, where HIF-1α inhibition significantly strengthened the cytotoxicity of methotrexate and cisplatin [66]. miR-199a has anti-tumour activity but is downregulated in patients with osteosarcoma. Intriguingly, it was found that HIF-1α is a direct target of miR-199a, and miR-199a exogenous expression resensitizes cisplatin-resistant cells to cisplatin by blocking HIF-1α expression [67]. SKA1 is a mitotic component responsible for accurate chromosomal segregation and cell division, which prevents the activation of drug resistance genes and enhances drug activity. However, hypoxia and the presence of HIF-1α downregulate SKA1 expression in osteosarcoma and promote chemoresistance. However, lentiviral-induced overexpression of SKA1 significantly reduced the expression of three drug resistance-related genes: multidrug resistance 1, multidrug resistance-associated protein 2, and glutathione S-transferase P, which help tumour cells to efflux the drugs out of the cells, both at the mRNA and protein levels. Simultaneously, it increases the sensitivity of osteosarcoma cells to drugs, particularly under hypoxic conditions (Table S3) [65]. Similarly, HIF-1α-induced upregulation of Mxd1 has been implicated in the development of drug resistance under hypoxia. Mxd1 inhibits Fas-mediated apoptosis in osteosarcoma cells by suppressing the expression of phosphatase and tensin homologue (PTEN). Additionally, it contributes to cisplatin resistance in hypoxic osteosarcoma cells by directly repressing the tumour suppressor gene PTEN, which activates the PI3K/AKT anti-apoptotic and survival pathways (Table S3) [68]. Therefore, HIF-1α promotes the activation of various oncogenes and pathways that promotion of osteosarcoma cells [69]. Therefore, regulation of these two major factors, RUNX2 and HIF-1α, may help overcome chemotherapeutic resistance, enhance overall and disease-free survival in patients with osteosarcoma, and stand out as an effective therapeutic approach.

### 3.5. Radiotherapy

Primary or acquired chemotherapy or radiation resistance leads to treatment failure and poor patients [69]. Radiotherapy is not a commonly used treatment for radioresistant osteosarcoma cells, as it is only partially effective for local osteosarcoma treatment [70,71]. However, osteosarcoma patients have a poor response to radiation therapy; until now, the precise mechanism underlying the development of radioresistance in osteosarcoma patients has not been adequately understood. However, hypoxic cells are more vulnerable to radioresistance [72], and osteosarcoma tumour tissues exhibit elevated HIF-1α expression [51]. To achieve this, Feng et al. proposed two phenomena underlying radioresistant osteosarcoma. First, radiation causes the production of free radicals in the DNA. Under normal conditions, oxygen can repair these radicals, resulting in reactive oxygen species (ROS) molecules that damage DNA and induce cellular death. However, this activity was suppressed in hypoxic cells. Second, active autophagy increases cell resistance to radiation by accelerating the clearance of cellular ROS products (Table S3) [73]. Thus, radioresistance is mainly mediated by HIF-1α. Moreover, RUNX2 may support this process by stabilizing and preventing HIF-1α production in tumour cells [23,24]. Furthermore, RUNX2 controls the expression of proteins that confer radioresistance to tumour cells. RUNX2/CXCR4/AKT/FOXA2 signalling, for example, regulates the expression of MRE11, a marker for radiation resistance and a predictor of a lower prognosis in patients, as in oral squamous cell carcinoma [74,75]. Additionally, RUNX2 and PI3K/AKT form a positive feedback loop that stimulates MMPs, VEGF, bone matrix proteins, bone-resorbing factors, and other genes related to invasion and metastasis, thus assisting in reprogramming cancer cells to undergo EMT [29]. This EMT transcription factor increases angiogenesis, prolonged proliferative signalling, evasion of growth inhibitory signals, and resistance to chemotherapy and radiation [29]. Thus, radiotherapy resistance in osteosarcoma may be caused by the combined action of RUNX2 and HIF-1α.

## 4. Conclusions

RUNX2 overexpression, whether at the protein or RNA level, is typically associated with aggressive tumour types, as has been reported in multiple studies. It inhibits the activation of both intrinsic and extrinsic apoptosis pathways and suppresses the activity of the p53 family. In contrast, RUNX2 prevents the degradation of HIF-1α, thereby stabilizing its expression. This stabilization of HIF-1α triggers the expression of hypoxia-responsive genes, leading to aberrant angiogenesis, shaping the tumour microenvironment and supporting the survival and maintenance of cancer stem cells. We propose that RUNX2 and HIF-1α complement each other in osteosarcoma progression through molecular crosstalk. Their presence in the VEGF promoter region suggests a complex relationship between HIF-1α and RUNX2, which has not been fully elucidated. However, the overexpression of both RUNX2 and HIF-1α significantly affects resistance to chemotherapy and radiotherapy. Therefore, we recommend that regulating RUNX2 and HIF-1α and addressing their molecular interactions is a crucial therapeutic target for osteosarcoma, which could enhance overall and disease-free survival. Nonetheless, further research is needed to gain a clearer understanding of how RUNX2 stabilizes HIF-1α, particularly under normal oxygen conditions. Most of these findings were derived from cell lines with limited patient data or in vivo validation. In conclusion, targeting the RUNX2–HIF-1α axis holds promise, but achieving clinical success requires deeper mechanistic insights and validation.

## Data Availability

No new data were created or analyzed in this study. Data sharing is not applicable to this article.

## Supplementary Materials

The following supporting information can be downloaded at: https://www.mdpi.com/article/10.3390/ijms26157642/s1.

## Abbreviations

The following abbreviations are used in this manuscript:

AMPK	adenosine monophosphate-activated protein kinase
EMT	Epithelial–mesenchymal transition
GLUT1	Glucose transporter 1
HIF-1α	hypoxia-inducible factor 1-alpha
MMP	Matrix metalloproteinase
mTOR	mammalian target of rapamycin
Mxd1	MAX dimerization protein 1
NOXA	phorbol-12-myristate-13-acetate-induced protein 1
PTEN	Phosphatase and tensin homologue
PHDs	Prolyl hydroxylase domain
PI3K/Akt	Phosphatidylinositol 3-kinase/protein kinase B
RUNX2	Runt-related transcription factor 2
SKA1	Spindle and kinetochore-associated complex subunit 1
VEGF	Vascular endothelial growth factor
pVHL	von Hippel Lindau tumour suppressor protein
ZEB	Zinc finger E-box-binding homeobox
