# Supplementary material for: Molecular Crosstalk Between RUNX2 and HIF-1α in Osteosarcoma: Implications for Angiogenesis, Metastasis, and Therapy Resistance
Source: Int J Mol Sci. 2025 Aug 7;26(15):7642. doi: 10.3390/ijms26157642 (PMC12347496; doi:10.3390/ijms26157642)
Supplement: Supplementary file 1 [file ijms-26-07642-s001.zip › ijms-3777668-supplementary.pdf]

Table S1 Studies depicting the molecular cross-talk of RUNX2 and HIF-1α

Studies	Findings	Significance	References
This study explores the relationship between HIF-1α and Runx2 in the control of VEGF gene expression and shows how canonical angiogenic and osteogenic transcription factors can function together to regulate an angiogenic signal.	HIF-1α and Runx2 physically and functionally interact to control VEGF synthesis. RUNX2 and HIF-1α were found to colocalize in specific nuclear regions of target cells and physically interact via the RUNX2 runt domain. Chromatin immunoprecipitation (ChIP) analysis further confirmed their interaction on the VEGF-A gene chromatin.	Increased the bone vascularization	[22]
This study focused on identifying the mediation of HIF-1α by Runx2 and its effect on the glycolytic metabolism of degenerative chondrocytes.	Silencing of RUNX2 inhibited HIF-1α expression and led to further impairment of glycolytic metabolism in degenerative chondrocytes. Therefore, it has been suggested that RUNX2 may regulate HIF-1α expression transcriptionally by binding directly to its promoter region.	Increased the glycolytic metabolism	[24]
The study focused on the intricate networking between HIF-1α and Runx2 in the growth plate.	Runx2 stabilized the HIF-1α via binding to oxygen-dependent degradation domain to block the interaction between the von Hippel-Lindau protein and HIF- 1α. This binding prevents the degradation of HIF-1α and helps in nuclear translocation.	Angiogenesis, suppress apoptosis, resulting in tumor progression	[23,25]

Investigated how melanoma bone metastases affect osteocytes using in vitro and in vivo models, RNA-seq, pathway analysis, and inhibitor studies.	Melanoma cells trigger ferroptosis in osteocytes via the HIF1α-HMOX1 axis, leading to iron overload, lipid peroxidation, and bone degradation.	Preserve bone integrity during treatment	[29]
RUNX2 and the PI3K/AKT axis reciprocal activation supports tumor progression	RUNX2 activation disrupt feedback loops which helps in sustaining VEGF/FAK signaling	Reduced metastasis and angiogenesis	[30]
The study mainly focused on identifying the effect of HIF-1α induction of hypoxia and activation of angiogenesis in osteosarcoma.	HIF-1α increases the VEGF level and Runx-2 expression. However, the HIF-1α silencing reduced the targeted protein VEGF, but it also lowered the Runx-2 expression.	Stimulates the angiogenesis	[40]
The aim of the study was to investigate the effect of FBW7 on the HIF-1α/VEGF pathway in IL-1β-induced degeneration of chondrocytes.	FBW7 negatively regulates the HIF-1α/VEGF pathway, suppresses RUNX2 and collagen I expression, and promotes expression of collagen II, aggrecan, and SOX-9.	Suppression of HIF-1α reduces VEGF and RUNX2 expression, indicating reduced angiogenesis and matrix degeneration.	[43]

Abbreviations: FAK: Focal Adhesion Kinase; HIF-1α: hypoxia inducible factor-1α; PI3K/AKT: Phosphatidylinositol 3-kinase / Protein kinase B; RUNX2: runt related transcription factor 2; VEGF: vascular endothelial growth factor.

Table S2 The downregulation and upregulation of genes under influence of RUNX2: ↑ Upregulated, ↓ Downregulated

**Marker expression**	**Functional Role**	**Genes**	**Significance**	**References**
Up-regulated	Angiogenesis	↑ VEGF	RUNX2 promotes the activation of angiogenesis precursor VEGF to promote tumor vascularization. Expression of VEGF supports tumor formation and enhanced vascularization, resulting in tumor growth. Additionally, VEGF expression has also been linked with invasion and metastasis.	[22-25]
	Metastasis	↑ MMP2<br>↑ MMP9<br>↑MMP13	Upregulation of MMP-2, MMP-9, and MMP-13 has been found in the presence of RUNX2. This MMP causes destruction of the extracellular matrix and promotes the metastasis and invasion in osteosarcoma.	[44-47]
	Apoptosis	↓Cytochrome C<br>↓ FADD<br>↓ Caspase-3<br>↓ Caspase-8<br>↓ Caspase-9	Runx2 prevents the activation of the intrinsic mitochondrial-mediated and extrinsic death receptor-mediated apoptotic pathways. It acts as	[16]

Down-regulated			the antagonist of the chemotherapy-induced apoptosis and prevents the osteosarcoma cells from apoptosis via suppressing the activation of caspases and death receptor ligands.	
	Apoptosis	↓p53	RUNX2 functions as a negative regulator of p53 following DNA damage. Elevated RUNX2 expression suppresses p53-targeted pro-apoptotic genes, including Bcl-2-associated protein x (Bax), NOXA, and the p53 upregulated modulator of apoptosis (PUMA). This suppression contributes to the inhibition of cell cycle arrest and apoptosis, thereby promoting drug resistance.	[13,59- 64]

Abbreviations: Bcl-2: B-cell lymphoma 2; MMP: matrix metalloproteinase; RUNX2: runt related transcription factor 2; VEGF: vascular endothelial growth factor; NOXA: phorbol-12-myristate-13-acetate-induced protein 1.

Table S3 The downregulation and upregulation of genes under influence of HIF-1α: ↑ Upregulated, ↓ Downregulated

Marker expression	Functional Role	Genes	Significance	References
Up-regulated	Glycolysis	↑Lactate dehydrogenase A<br>↑GLUT1<br>↑GLUT4<br>↑Pyruvate dehydrogenase kinase 1 and 4	HIF-1α promotes the glycolysis and adaptation of cells to hypoxia through increased glucose uptake. Elevated Lactate dehydrogenase A causes decreased oxidative mitochondrial function and maintains cell survival under hypoxic conditions and promotes tumor progression.	[19,23]
	Angiogenesis	↑ VEGF	HIF-1α enhances VEGF expression, which drives angiogenesis and tumor progression. Additionally, by upregulating VEGF-A, HIF-1α promotes osteosarcoma cell invasion, contributing to a poor prognosis in osteosarcoma patients.	[22,25,35,39,40,43]
		↑MMP2<br>↑MMP9	HIF-1α promotes the transcription of MMPs to degrade the extracellular matrix. MMPs also upregulate the VEGF	[51,53]

			expression in tumor cells and promote the interaction of VEGF and its receptors. Thus, elevated MMPs result in the promotion of metastasis, invasion, and angiogenesis.	
	EMT	↑Neural-cadherin, ↑Vimentin, ↑Snail, ↑ Zinc finger E-box-binding homeobox ↑Twist-related protein	HIF-1α stimulates the expression of N-cadherin, vimentin, Snail, Zinc finger E-box-binding homeobox, and Twist-related protein, driving epithelial-mesenchymal transition (EMT). This process leads to loss of epithelial polarity, weakening of cell junctions, and reorganization of cytoskeletal proteins, thereby promoting tumor invasion and metastasis.	[51,53]
	Apoptosis and Resistance	↑ Mxd1	HIF-1α increases the expression of Mxd1, which inhibits Fas-induced apoptosis in osteosarcoma cells by suppressing the tumor suppressor gene PTEN.	[68]
		↑ Light chain - 3I ↑ Light chain - 3II	HIF-1α increased the autophagy markers light chain 3-I and 3-II. Higher expression of these markers	[73]

			results in the radio resistance.	
	EMT	↓epithelial-cadherin	The hypoxia microenvironment down- regulated the expression of epithelial-cadherin. This promotes the EMT, a crucial step for metastasis.	[51-53]
Down-regulated	Apoptosis and Resistance	↓ SKA1	HIF-1α downregulates the activity of the SKA1 tumor suppressor gene. Downregulation of SKA1 stabilizes the drug-resistance related genes: Multidrug resistance 1, Multidrug resistance associated protein 2, and Glutathione S-transferase P, which help tumor cells to efflux the drugs out of the cells both at the mRNA and protein levels. This causes the resistance of osteosarcoma cells to chemotherapeutic agents under hypoxic conditions.	[65]
		↓PTEN	HIF-1α downregulates the activity of the PTEN tumor suppressor gene. PTEN downregulation	[68]

			promotes the activation of PI3K/AKT, which provides antiapoptotic effects and survival benefits to osteosarcoma cells.	

Abbreviations: EMT: epithelial–mesenchymal transition; HIF-1α: hypoxia inducible factor-1α; Mxd1: MAX dimerization protein 1; PTEN: Phosphatase and Tensin homolog; PI3K/AKT: Phosphatidylinositol 3-kinase / Protein kinase B; SKA1: spindle and kinetochore- associated complex subunit 1; VEGF: vascular endothelial growth factor.
